# CXCR5 and TIM-3 expressions define distinct exhausted T cell subsets in experimental cutaneous infection with *Leishmania mexicana*

**DOI:** 10.3389/fimmu.2023.1231836

**Published:** 2023-08-25

**Authors:** Mariana Diupotex, Jaime Zamora-Chimal, Julián A. Gajón, Laura C. Bonifaz, Ingeborg Becker

**Affiliations:** ^1^ Unidad de Investigación en Medicina Experimental, Facultad de Medicina, Universidad Nacional Autónoma de México, Ciudad de México, Mexico; ^2^ Unidad de Investigación Médica en Inmunoquímica, Hospital de Especialidades, Centro Médico Nacional Siglo XXI, Instituto Mexicano del Seguro Social, Ciudad de México, Mexico; ^3^ Coordinación de Investigación en Salud, Centro Médico Nacional Siglo XXI, Instituto Mexicano del Seguro Social, Ciudad de México, Mexico

**Keywords:** cutaneous leishmaniasis, chronic infection, PD-1, T-cell exhaustion, exhausted subpopulations, progenitor exhausted cells, terminally-differentiated cells

## Abstract

T-cell exhaustion is a key stage in chronic infections since it limits immunopathology, but also hinders the elimination of pathogens. Exhausted T (Tex) cells encompass dynamic subsets, including progenitor cells that sustain long-term immunity through their memory/stem like properties, and terminally-differentiated cells, resembling the so-called Tex cells. The presence of Tex cells in chronic leishmaniasis has been reported in humans and murine models, yet their heterogeneity remains unexplored. Using flow cytometry, we identified Tex cells subtypes based on PD-1, CXCR5 and TIM-3 expressions in draining lymph nodes (dLNs) and lesion sites of C57BL/6 mice infected with *L. mexicana* at 30-, 60- and 90-days post-infection. We showed that infected mice developed a chronic infection characterized by non-healing lesions with a high parasite load and impaired Th1/Th2 cytokine production. Throughout the infection, PD-1^+^ cells were observed in dLNs, in addition to an enhanced expression of PD-1 in both CD4^+^ and CD8^+^ T lymphocytes. We demonstrated that CD4^+^ and CD8^+^ T cells were subdivided into PD-1^+^CXCR5^+^TIM-3^-^ (CXCR5^+^), PD-1^+^CXCR5^+^TIM-3^+^ (CXCR5^+^TIM-3^+^), and PD-1^+^CXCR5^-^TIM-3^+^ (TIM-3^+^) subsets. CXCR5^+^ Tex cells were detected in dLNs during the whole course of the infection, whereas TIM-3^+^ cells were predominantly localized in the infection sites at day 90. CXCR5^+^TIM-3^+^ cells only increased at 30 and 60 days of infection in dLNs, whereas no increase was observed in the lesions. Phenotypic analysis revealed that CXCR5^+^ cells expressed significantly higher levels of CCR7 and lower levels of CX3CR1, PD-1, TIM-3, and CD39 compared to the TIM-3^+^ subset. CXCR5^+^TIM-3^+^ cells expressed the highest levels of all exhaustion-associated markers and of CX3CR1. In agreement with a less exhausted phenotype, the frequency of proliferating Ki-67 and IFN-γ expressing cells was significantly higher in the CXCR5^+^ subset within both CD4^+^ and CD8^+^ T cells compared to their respective TIM-3^+^ subsets, whereas CD8^+^CXCR5^+^TIM-3^+^ and CD8^+^TIM-3^+^ subsets showed an enhanced frequency of degranulating CD107a^+^ cells. In summary, we identified a novel, less-differentiated CXCR5^+^ Tex subset in experimental cutaneous leishmaniasis caused by *L. mexicana*. Targeting these cells through immune checkpoint inhibitors such as anti-PD-1 or anti PD-L1 might improve the current treatment for patients with the chronic forms of leishmaniasis.

## 1 Introduction

Cutaneous leishmaniasis (CL) is the most prevalent form of human leishmaniasis caused by vector-borne protozoan parasites, with an estimated 200,000 new cases reported annually worldwide (1). *Leishmania* (L.) *mexicana* is the main species responsible for CL in Mexico, causing several clinical presentations that range from self-healing localized cutaneous leishmaniasis (LCL) to chronic diffuse cutaneous leishmaniasis (DCL) (2). The former is characterized by a single or limited number of ulcerative skin lesions with few parasites on the ulcer border, whereas the latter is associated with multiple disseminated non-ulcerative nodular lesions with abundant parasitic infiltration and negative delayed-type hypersensitivity skin reaction to leishmanial antigens (3).

A protective immune response against *Leishmania* infection relies mainly on the establishment of a CD4^+^ Th1-type immune response, which leads to parasite killing within host phagocytic cells by the production of reactive oxygen species (ROS) and nitric oxide-free radicals (NO). Conversely, susceptibility to CL is related to a dominant CD4^+^ Th2 response that inhibits leishmanicidal functions of host cells and induces arginase activity, which results in parasite survival and proliferation (4, 5). CD8^+^ T cells also provide a protective response through IFN-γ production, which amplifies the early differentiation of CD4^+^ Th1 cells via IL-12 signaling and induces macrophage activation (6). One important mechanism underlying impaired T-cell immunity during either active or chronic human leishmaniasis is the substantial loss of effector functions in a process termed exhaustion (7–12).

T-cell exhaustion represents a functional hyporesponsive state, occurring as a physiological adaptation to prevent excessive immune-mediated tissue damage in chronic infections and malignancies (13–15). Exhausted T (Tex) cells are defined by an altered transcriptional program, distinct from effector or memory T cells, with restrained proliferative and effector functions. These cells show a maintained up-regulation of multiple inhibitory immune checkpoint receptors or ectonucleotidases, such as programmed cell death protein 1 (PD-1), T cell immunoglobulin and mucin domain-containing protein 3 (TIM-3), cytotoxic T lymphocyte-associated antigen 4 (CTLA-4), lymphocyte-activation gene 3 (LAG-3), T-cell immunoreceptor with immunoglobulin and ITIM domain (TIGIT), the cluster of differentiation (CD) 160 (CD160), 2B4/CD244, and CD39 (14, 15).

PD-1 regulates T-cell activation by engagement with its ligands PD-L1 and PD-L2, which are expressed by antigen-presenting cells (APCs) and stromal cells (15). Under physiological conditions, the PD-1/PD-L1 pathway modulates the establishment and maintenance of peripheral tolerance (16). However, during long-term antigen exposure, high PD-1 expression correlates with T-cell exhaustion (17). Targeting this pathway in current cancer immunotherapy has emerged as a successful strategy for the treatment of several hematological and solid malignancies, as it reinvigorates T-cell expansion and effector functions (18). Monoclonal antibodies (mAbs), such as anti-PD-1 or anti-PD-L1, have emerged as promising therapeutic agents for the treatment of parasitic infections in humans (19).

Recent advances have demonstrated that Tex cells constitute a dynamic network of subsets with distinct transcriptional, phenotypic and functional properties (20). Accordingly, Tex cells include two main subgroups: progenitor-like exhausted cells, characterized by the co-expression of PD-1, CXC-chemokine receptor 5 (CXCR5) and transcriptional regulator T cell factor 1 (TCF1), and terminally-differentiated cells, which exhibit the co-expression of several exhaustion-associated markers, including PD-1 and TIM-3, but lack the expression of CXCR5 and TCF1 (21–25). Progenitor-like Tex cells play an essential role in sustaining long-term immunity, as they have the ability to continuously self-renew and give rise to transitory exhausted progeny with enhanced cytolytic activity, before undergoing to terminally-differentiated Tex cells (21–24). By contrast, terminally-differentiated Tex cells exhibit greater dysfunctionality as they lack self-renewal and proliferative capacity, but retain a degree of cytotoxicity (25–27). Interestingly, progenitor-like cells mediate the response to PD-1 immunotherapy by undergoing a proliferative burst, leading to the control of viral replication and tumor growth (21, 22, 27). Additionally, TCF1^+^ tumor-infiltrating lymphocytes have been associated with a favorable prognosis in metastatic melanoma patients treated with immune checkpoint inhibitors, suggesting their potential role as a predictive biomarker (28, 29). Accordingly, identifying different Tex subsets during CL would represent a significant progress in the understanding of infection outcomes, and could also enable the development of new targeting therapies for patients with DCL.

In this study, therefore, we first examined the infection outcome by *L. mexicana* in C57BL/6 mice subcutaneously infected with 1x10^5^ stationary-phase promastigotes. Further, we identified PD-1^+^ Tex subsets within both CD4^+^ and CD8^+^ T-cell compartments based on CXCR5 and TIM-3 expressions in lymph nodes and infection sites. Finally, we comparatively analyzed the phenotypical and functional profile of Tex subsets during the chronic phase of the infection.

## 2 Materials and methods

### 2.1 Mice and ethical statement

Female C57BL/6 mice, aged eight to ten weeks, were obtained from the breeding stock of the pathogen-free facility at the Unidad de Investigación en Medicina Experimental, Facultad de Medicina, Universidad Nacional Autónoma de México (UNAM). Animals were selected randomly and housed into microisolator cages with unrestricted access to food and water. All experiments were conducted following the guidelines for Animal Health NOM-062-ZOO-1999 and were approved by the Comité Interno para el Cuidado y Uso de Animales de Laboratorio, UNAM (Approval No. 123-2019/038-CIC-2019 and 063-2020/022-CIC-2020).

### 2.2 Chemicals, reagents and antibodies

L-glutamine (Cat. G3126), penicillin/streptomycin (Pen/Strep; Cat. P0781), collagenase (Cat. C5138), trypan blue (Cat. T8154), ionomycin calcium salt (Cat. 10634), phorbol 12-myristate 13-acetate (PMA; Cat. P8139), concanavalin A (ConA; Cat. C0412), DAPI (Cat. D9542), bovine serum albumin (BSA; Cat. A2153), Triton X-100 (Cat. 9036-19-5), and Trisodium citrate dihydrate (Cat. 6132-04-3) were purchased from Sigma-Aldrich (St. Louis, MO, USA). Sodium bicarbonate was obtained from J.T. Baker (Center Valley, PA, USA; Cat. 3506-01). β-mercaptoethanol (Cat. 21985-023), CFDA-SE (Cat. C1157), CountBright™ absolute counting beads (Cat. C36950), and Hoechst 33258 Pentahydrate (bis-Benzimide) (Cat. H3569) were purchased from Gibco or Invitrogen, subsidiaries of Thermo Fisher Scientific (Waltham, MA, USA). VectaShield Antifade Mounting Medium was purchased from Vector Laboratories (Burlingame, CA, USA; Cat. H-1000-10). Perm/Wash Buffer 10X (Cat. 421002) and monensin (Cat. 420701) were obtained from BioLegend (San Diego, CA, USA). Ghost Dye™ Violet 510 was purchased from Cytek, Tonbo Biosciences (San Diego, CA, USA). All fluorescence-labeled antibodies, except otherwise mentioned, were purchased from BioLegend: PerCP/Cyanine5.5 anti-mouse CD3ε (Cat. 100328), violetFluor 450 anti-mouse CD3 (Cytek, Tonbo Biosciences; Cat. 75-0032), FITC anti-mouse CD4 (Cat. 100406), PE/Cy7 anti-mouse CD4 (Cytek, Tonbo Biosciences; Cat. 60-0042), PE anti-mouse CD8a (Cat. 100708), Alexa Fluor 488 anti-mouse CD8a (Cat. 100723), PE/Cy7 anti-mouse PD-1 (Cat. 135216), PE anti-mouse PD-1 (Cat. 135206), APC anti-mouse PD-1 (Cat. 109112), APC anti-mouse CXCR5 (Cat. 145506), APC/Fire 750 anti-mouse TIM-3 (Cat. 134018), PE/Dazzle 594 anti-mouse TIM-3 (Cat. 134013), Brilliant Violet 785 anti-mouse CCR7 (Cat. 120127), PE anti-mouse CX3CR1 (Cat. 149006), PE/Dazzle 594 anti-mouse CD39 (Cat. 143812), Pacific Blue anti-mouse Ki-67 (Cat. 652422), FITC anti-mouse CD107a (Cat. 121606), and Alexa Fluor 488 anti-mouse IFN-γ (BD Biosciences, San Jose, CA, USA; Cat. 557724).

### 2.3 Parasite culture and lysate antigen preparation

Promastigotes of *L. mexicana*, strain MHOM/MX/2011/Lacandona, derived from amastigotes isolated from infected mice were cultured in medium 199 with Hank´s salt and L-glutamine, pH 7.2 (Sigma-Aldrich), supplemented with 2 mM L-glutamine, 0.35g/L sodium bicarbonate, and 10% of heat-inactivated fetal bovine serum at 26°C (FBS; Gibco, Thermo Fisher Scientific). Lysate antigen was obtained from axenic culture of stationary-phase promastigotes (pLAg) subjected to 3 rapid freeze-thaw cycles at -70°C, alternated with 3 cycles of bath sonication for 10 min at 4°C. Parasite lysis was confirmed by visual inspection using an optical microscopy. The total concentration of proteins within promastigotes lysate was determined by Bradford method (Sigma-Aldrich) with BSA used as the standard. Protein concentration was adjusted to 1 mg/mL and stored at -70°C until used.

### 2.4 Infection and parasite quantification

Mice were subcutaneously inoculated into the right hind footpad with 1x10^5^ stationary-phase promastigotes of *L. mexicana*. Lesion size was monitored by measuring the thickness with a digital micrometer caliper once a week during 12 weeks. The parasite burden was evaluated in digital images of infected footpad sections stained with hematoxylin and eosin (H&E) and visualized using a light microscope with an AxioCam MRc5 camera (Carl Zeiss, Oberkochen, Germany). FIJI ImageJ Software 1.47v (ImageJ NIH, Bethesda, Maryland, USA) was used to determine the number of amastigotes observed in 8 pictures taken at 40x magnification, corresponding to 1 mm^2^.

### 2.5 Isolation of lymph nodes and footpad cells

Popliteal draining lymph nodes (dLNs) were individually homogenized with a syringe plunger, filtered through a 40 μm-pore-size cell strainer and washed with 1X phosphate buffer saline (PBS), pH 7.4. For enzymatic isolation of dermal cells, footpads were minced into small pieces and put into a 24-well cell culture plate containing RPMI-1640 medium, pH 7.2 (Sigma-Aldrich; Cat. R8758) with 10% FBS and 0.5 mg/mL collagenase, followed by 90 min of incubation at 37°C. Following enzymatic digestion, the remaining tissues were homogenized, filtered and washed as above described. Both isolated dLNs and footpad cells were counted for viability determination using trypan blue exclusion assay. All *in vitro* assays were performed with complete RPMI-1640 medium supplemented with 10% FBS, 55 μM β-mercaptoethanol, and 1% Pen/Strep (10,000 U/mL, 10 mg/mL).

### 2.6 Cytokine quantification in culture supernatants

Isolated dLNs cells were plated at a density of 1x10^6^ cells/well in 96-flat bottom plate and incubated with 10 μg/mL pLAg for 96 h at 37°C. After incubation, the cell culture was centrifugated to remove any remaining cellular debris, and the resulting supernatant was stored at -70°C until used. Th1/Th2 cytokine (IFN-γ, TNF-α, IL-2, IL-4, IL-5, IL-6, IL-10, and IL-13) levels were determined by LEGENDplex beads-based immunoassay (BioLegend), according to the manufacturer´s instructions. Briefly, supernatant samples diluted 1:2 were mixed with 25 μL assay buffer, 25 μL mixed capture beads (1X) and 25 μL detection antibodies in 96-well polypropylene V-bottom plates, and incubated for 3 h at room temperature. Thereafter, 25 μL streptavidin-phycoerythrin (SA-PE) was added and incubated for 30 min. Finally, beads were washed with 1X Wash Buffer and resuspended for FACS analysis. Data were collected using a FACS Canto II flow cytometer (BD Bioscience, San Jose, CA, USA), and analyzed with LEGENDplex Data Analysis Software. Cytokine concentration was determined using seven 4-fold serial dilutions of standard cocktail of cytokines provided by the kit.

### 2.7 *In vitro* assays for functional characterization

For proliferation analysis, freshly isolated dLNs cells were seeded into 96-flat bottom plates at a concentration of 1x10^6^ cells/well containing complete RPMI-1640 medium, and then stimulated with 5 μg/mL ConA for 96 h at 37°C. Thereafter, Ki-67 expression was determined by flow cytometry. For intracellular cytokine detection and degranulation assay, 1x10^6^ cells/well were seeded in 96-flat bottom plates and stimulated with 50 ng/mL PMA and 1 μg/mL ionomycin for 2 h at 37°C. Further, cells were incubated with 2 μM monensin and degranulation marker anti-CD107a (1:100) during 4 h at 37°C. After stimulation, cells were harvested and labeled to evaluate IFN-γ expression and degranulation by flow cytometry.

### 2.8 Flow cytometry

Cell surface staining was performed by incubating the freshly isolated or stimulated cells derived from lymph nodes or infected footpads with fluorochrome-conjugated mAbs for T lymphocytes classification (anti-CD3, anti-CD4 and anti-CD8), exhaustion-associated receptors (anti-PD-1, anti-TIM-3 and anti-CD39), lymphoid tissue-homing receptors (anti-CXCR5 and anti-CCR7) and peripheral tissue-homing receptor (anti-CX3CR1) at a concentration of 1:100 diluted in 1X PBS for 20 min at 4°C. Dead cells were excluded using Ghost Dye Violet 510 (1:1000) or DAPI viability dye added concurrently with surface antibodies. After incubation, stained cells were centrifugated at 400 x g for 5 min at 4°C, and washed twice with PBS. Equivalent numbers (0.15x10^5^) of CountBright beads were added to each sample prior to acquisition and at least 5,000 beads events were recorded to determine the absolute cell count as per the manufacturer´s instructions. For intracellular staining, extracellular labeling was performed as described above. After the final wash step in PBS, cells were fixed in 2% paraformaldehyde for 15 min at 4°C and permeabilized twice with 1X Perm/Wash buffer. After that, cells were stained with fluorochrome-conjugated mAbs anti-IFN-γ and anti-Ki-67 diluted 1:100 in Perm/Wash buffer for 30 min at 4°C. Following intracellular labelling, cells were centrifugated at 400 x g for 5 min at 4°C, washed once with Perm/Wash, and resuspended in PBS for FACS analysis. All data were collected using a FACS Canto II, except for the phenotypic profile data of Tex subsets, which were acquired in a Cytek Aurora Spectral cytometer (Cytek Biosciences, Fremont, CA, USA). At least 10,000 events corresponding to the area of lymphocytes (SSC-H vs. FSC-H) were recorded for each sample. FSC files were analyzed by FlowJo v.10 Software (Treestar, Ashland, OR, USA). Mean fluorescence intensity (MFI) was calculated by averaging the median values of each data set. The gating strategy and fluorescence minus one (FMO) controls for identifying Tex subsets, are presented in **Supplementary Figure 1**.

### 2.9 Immunofluorescence

Footpad tissues were fixed with 4% formaldehyde for 24-72 h, embedded on paraffin blocks, and sectioned into 3-μm thick slices deposited onto positively charged glass slides. Tissues slides were deparaffinized by incubation for 45 min at 60-65°C prior to xylene incubation for 5 min and rehydrated with a graded series of xylene/ethanol solvents (Xylene50%/Et50%, Et100%, Et80%, Et80%, Et50%, H_2_0). Heat-induced antigen retrieval was performed using 10 mM sodium citrate buffer, pH 6.0 for 20 min at 90°C. Thereafter, the slides were blocked and permeabilized in 1% BSA, 5% horse serum, and 0.3% Triton X-100 in 1X PBS for 2 h. Following permeabilization, samples were incubated overnight with fluorochrome-conjugated mAbs anti-CD3, anti-CD4, anti-CD8, anti-PD-1, anti-CXCR5, or anti-TIM-3 at 1:50 concentration. The nuclei were counterstained with Hoechst 33258 (1:2000) for 10 min, and the slides were mounted with VectaShield Mounting Medium. Micrographs were obtained on a Nikon Ti Eclipse inverted confocal microscope (Nikon Corporation, Minato, Tokyo, Japan) equipped with an A1 imaging system controlled by the proprietary software NIS Elements v.4.50 (Melville, NY, USA). Imaging was performed using a 20x objective lens, and magnification was performed either at 3x during image acquisition or with digital zoom. Confocal microscopy analysis of immunofluorescence was carried out using FIJI ImageJ Software 1.47v.

### 2.10 Statistical analysis

Prior to analysis, data normality and variance homogeneity were verified using Shapiro-Wilk and Levene´s tests, respectively, by IBM SPSS Statistics 29.0.1.0 (IBM, Armonk, NY). Then, statistical significance was performed by non-parametric, two-tailed Mann-Whitney U-test for unpaired samples. *P*-values of < 0.05 indicate the significant differences between groups. Statistical analysis was performed using Prism v.8.0.1 Software (GraphPad Software Inc., CA, USA).

## 3 Results

### 3.1 Experimental infection with *L. mexicana* leads to persistent lesions with high parasite load and impaired Th1/Th2 cytokine production

**Figure 1:**
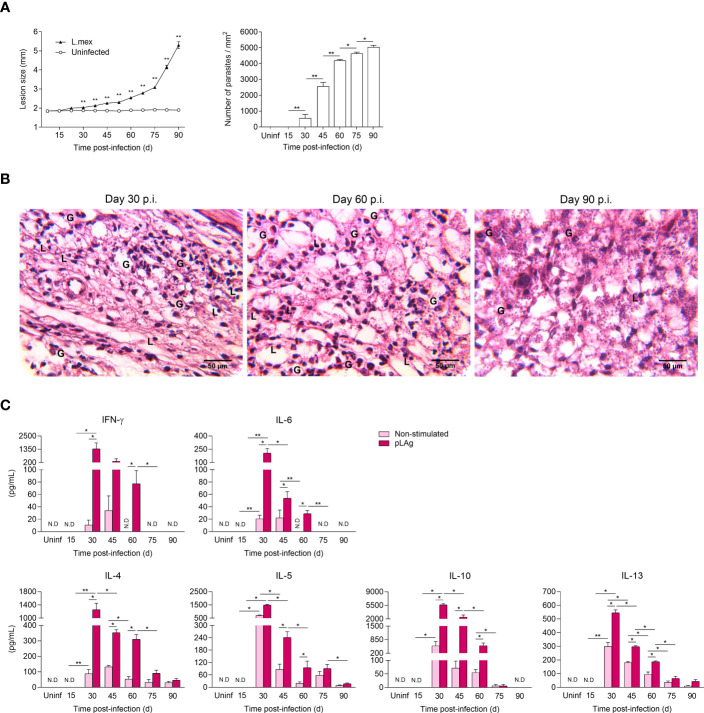
Kinetic evaluation of lesion size, tissue parasitism, histopathological changes and Th1/Th2 cytokine production during cutaneous infection with *L. mexicana* in C57BL/6 mice. **(A)** Cutaneous lesion development and intralesional parasite numbers of C57BL/6 mice infected with 1x10^5^ stationary-phase promastigotes. The data represent two independent experiments and are presented as the mean value ± SD (n=5). **(B)** Representative hematoxylin and eosin staining of footpad tissue sections at indicated times. **(C)** Cytokine production in the supernatants of draining lymph node cells, either non-stimulated or stimulated with pLAg (10 μg/mL) during 96 h. Data represent the mean value ± SD (n=3). Mann-Whitney U-test, where *p<0.05; **p<0.01. Uninf, uninfected; d, days; G, granulocytes; L, lymphocytes; N.D, not detected.

In order to characterize the experimental infection of *L. mexicana*, lesion swelling, parasite burden, and inflammatory infiltrate were evaluated at indicated time points in C57BL/6 mice. Infected mice developed progressive non-healing lesions after 30 days post-infection (p.i.). Notably, during the early stage of the infection (30-60 days p.i.), the lesion sizes increased gradually, followed by a dramatic increase in the chronic stage ranging from 75 to 90 days. Parasite load also increased gradually along the course of the infection, reaching a significantly peak load of ~5,046 parasites/mm^2^ at 90 days p.i. (**Figure 1A**). Histopathological analyses also showed numerous granulocytes and lymphocytes infiltrating the infected paws at 30 and 60 days after infection. However, at the chronic stage (90 days p.i.), footpad lesions exhibited a lower infiltration of these immune cells, accompanied by a higher number of parasites (**Figure 1B**). Overall, these findings indicate that C57BL/6 mice fail to resolve *L. mexicana* infection, but instead develop a chronic infection that can become increasingly severe over time. To ascertain the antigen-specific response during the course of the infection, supernatants of dLNs cells stimulated with pLAg were analyzed for cytokine quantification by flow cytometry. Increased levels of IFN-γ, IL-6, IL-4, IL-5, IL-10, and IL-13 were significantly detected as early as 30 days p.i., reaching their maximum production at this time point. No TNF-α and IL-2 production was observed at any analyzed time (data not shown). Intriguingly, after 30 days of infection, the levels of all cytokines decreased significantly over time, yet anti-inflammatory cytokines (IL-4, IL-5, IL-10 and IL-13) decreased to a lesser extent. No detectable levels of IFN-γ and IL-6 were found during the chronic phase of the infection (90 days p.i.). Additionally, there was a significant decrease production of 13.8-fold in IL-4, 21.3-fold in IL-5, and 12.6-fold in IL-13 at day 90, compared to the highest levels observed at 30 days of infection. It is noteworthy, that no IL-10 production was observed after 75 days p.i. (**Figure 1C**). Taken together, these data demonstrate that the production of Th1/Th2 cytokines by dLNs in response to lysate antigen decreased over time as the infection progresses to a chronic stage.

### 3.2 PD-1^+^ cells and the expression intensities of PD-1 are up-regulated on CD4^+^ and CD8^+^ T cells

**Figure 2:**
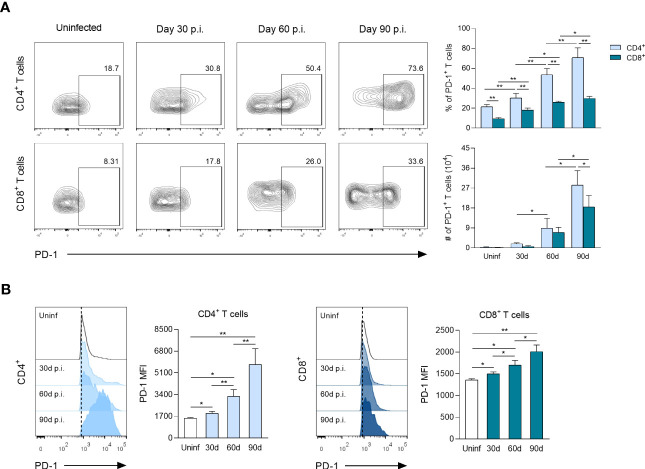
PD-1 surface expression on CD4^+^ and CD8^+^ T cells following *L. mexicana* infection. **(A)** Representative flow cytometry analysis and summary data of the frequency (%) and absolute cell numbers (#) of CD4^+^PD-1^+^ and CD8^+^PD-1^+^ T-cell compartments derived from draining lymph nodes (dLNs) of infected mice at indicated times. **(B)** Representative histograms and summary data of mean fluorescent intensity (MFI) demonstrating PD-1 expression on CD4^+^ and CD8^+^ T cells from dLNs at indicated times. Data are representative of two independent experiments and are presented as the mean ± SD (n=5). Mann-Whitney U-test, where *p<0.05; **p<0.01. Uninf, uninfected; d, days; p.i., post-infection.

To determine the kinetics of PD-1^+^ T cells and expression levels of PD-1 in response to *L. mexicana* infection, CD4^+^ and CD8^+^ T cells derived from dLNs were analyzed by flow cytometry. The frequency (%) and absolute cell count (#) of PD-1^+^ cells among CD4^+^ and CD8^+^ T cells showed a gradual fold-increase over infection time (**Figure 2A**). Thus, the frequency of PD-1^+^ cells at 30, 60 and 90 days p.i. increased, respectively, by 1.3, 2.4 and 3.3 times within CD4^+^ T cells, whereas CD8^+^ T-cells increased 1.9, 2.8 and 3.2 times, respectively, both compared to uninfected controls. Interestingly, the frequency of PD-1^+^ cells was significantly higher within CD4^+^ T cells throughout the course of the infection. However, the absolute cell numbers of PD-1^+^ cells were only significantly higher during the chronic phase (90 days p.i.) (**Figure 2A**). FACS analysis also showed that PD-1 MFI was significantly up-regulated on CD4^+^ and CD8^+^ T cells of infected mice in comparison with uninfected controls, showing a progressive increase throughout the infection time (**Figure 2B**). The expression kinetics further revealed differences in the magnitude of PD-1 MFI between CD4^+^ and CD8^+^ T cells. Thus, up-regulated PD-1 MFI occurred especially on CD4^+^ T cells, reaching a 1.3, 2.1 and 3.7-fold increase at days 30, 60 and 90 after infection, respectively, as compared to uninfected controls. In contrast, up-regulated PD-1 MFI on CD8^+^ T cells only reached a 1.1, 1.2 and 1.4-fold increase, respectively, at the same time points (**Figure 2B**). Overall, these findings suggest that an increasing number of Tex cells (PD-1^+^) are differentiated during the chronic stage of experimental infection with *L. mexicana* in response to tissue parasitism, highlighting that a greater number of CD4^+^ T cells exhibit an exhausted phenotype.

### 3.3 Tex cells display heterogeneous phenotypes based on CXCR5 and TIM-3 expressions

**Figure 3:**
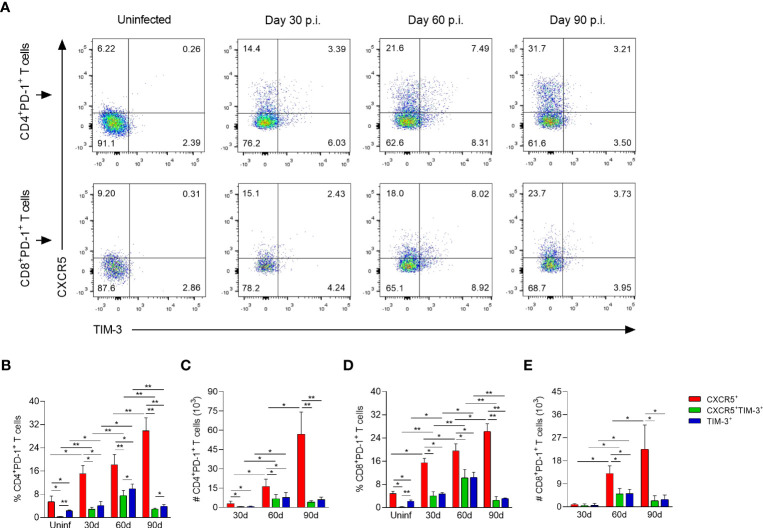
CD4^+^ and CD8^+^ Tex subsets in popliteal draining lymph nodes of *L. mexicana*-infected mice. **(A)** Representative flow plots of CXCR5 and TIM-3 expression on CD4^+^PD-1^+^ and CD8^+^PD-1^+^ T cells at indicated times. Bar graphs showing the frequency **(B, D)** or absolute cell numbers **(C, E)** of Tex subsets within CD4^+^PD-1^+^ and CD8^+^PD-1^+^ T-cell compartments. Summary data shows the mean ± SD of two independent experiments (n=5). Mann-Whitney U-test, where *p<0.05; **p<0.01. Uninf, uninfected; d, days; p.i., post-infection.

To identify the heterogeneity and dynamic changes of CD4^+^ and CD8^+^ Tex cells during *L. mexicana* infection, dLNs cells of infected mice were co-stained with CXCR5 and TIM-3, and subjected to a flow cytometry time-course analysis. Using these markers, three distinct subsets of Tex cells emerged within CD4^+^PD-1^+^ and CD8^+^PD-1^+^ compartments: CXCR5^+^, CXCR5^+^TIM-3^+^ and TIM-3^+^. Of note, this pattern was present throughout the infection, although different subsets were enriched during the early (30-60 days p.i.) vs chronic (90 days p.i.) phase (**Figure 3A**). Thus, the frequencies of CXCR5^+^ subset of CD4^+^ and CD8^+^ Tex cells showed a significantly gradual increase over time, comprising 15 to 30% of overall exhausted subsets, depending on the time of analysis (**Figures 3B, D**). The absolute cell numbers of this subset within both types of T lymphocytes also increased during the infection times. Interestingly, the number of CD4^+^CXCR5^+^ cells was significantly (2.5-fold) higher than that of CD8^+^CXCR5^+^ T cells in the chronic phase (**Figures 3C, E**). On the other hand, the frequencies of CXCR5^+^TIM-3^+^ cells within both CD4^+^ and CD8^+^ T cells reached comparable values at 30 (2-4%) and 60 (7-10%) days after infection. Similarly, CD4^+^TIM-3^+^ and CD8^+^TIM-3^+^ cells achieved analogous percentages at 30 (4-5%) and 60 (10-11%) days of infection. Notably, the frequencies of both CXCR5^+^TIM-3^+^ and TIM-3^+^ subsets within CD4^+^ and CD8^+^ T-cell compartments significantly declined at day 90 during the chronic phase (**Figures 3B, D**). The absolute cell count of CXCR5^+^TIM-3^+^ and TIM-3^+^ cells of CD4^+^ and CD8^+^ T-cell compartments significantly increased from day 30 to 60, and remained high at least until day 90 of infection (**Figures 3C, E**). In summary, these data demonstrate three major populations of Tex cells during experimental infection with *L. mexicana*, including the previously defined CXCR5^+^ and TIM-3^+^ subsets, and also one distinct subset characterized by CXCR5 and TIM-3 co-expression. These results also highlight that the CXCR5^+^ subsets are the most abundant cells draining the lymph nodes from the infection sites during the course of the infection, whereas CXCR5^+^TIM-3^+^ and TIM-3^+^ cells decline during the chronic phase.

### 3.4 Tex subsets exhibit a distinct phenotypic profile during the chronic phase of the infection

**Figure 4:**
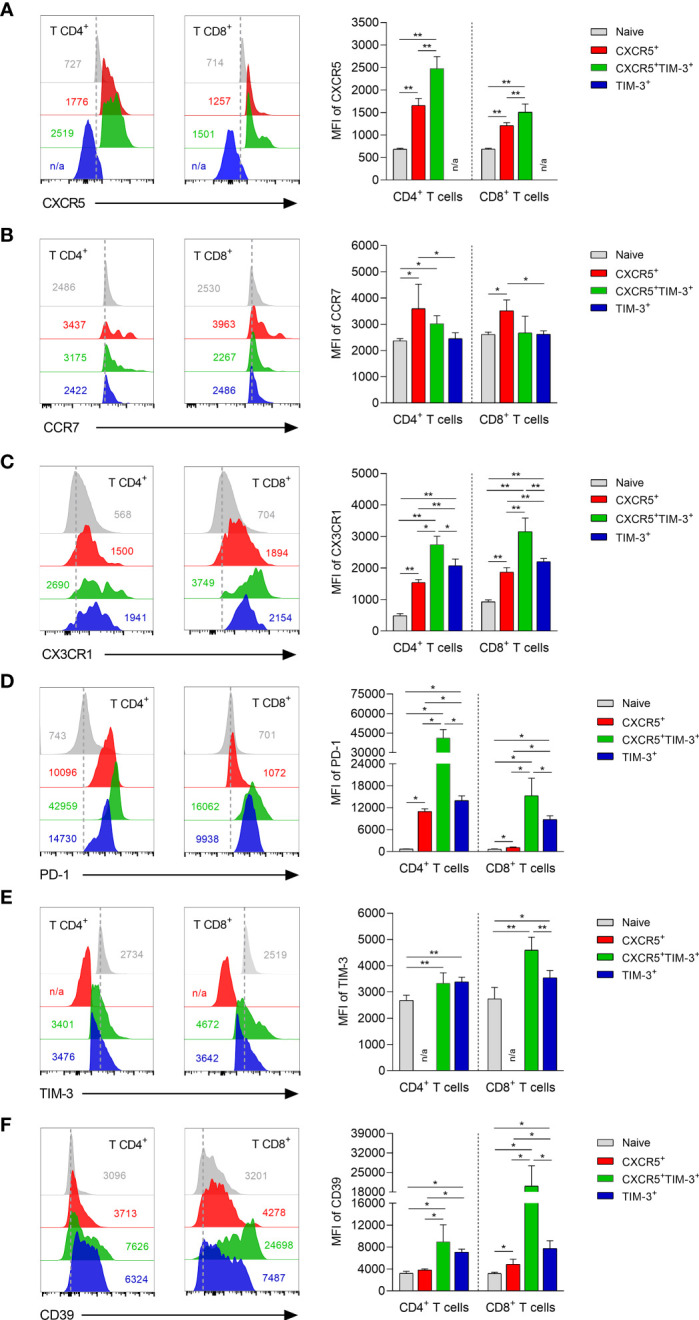
Phenotyping characterization of CXCR5^+^, CXCR5^+^TIM-3^+^ and TIM-3^+^ subsets during the chronic *L. mexicana* infection. Representative histograms and summary data showing the mean fluorescence intensity (MFI) of CXCR5 **(A)**, CCR7 **(B)**, CX3CR1 **(C)**, PD-1 **(D)**, TIM-3 **(E)** and CD39 **(F)** on CD4^+^PD-1^+^ and CD8^+^PD-1^+^ T-cell compartments derived from draining lymph nodes of chronically infected mice (90 days after infection). CD3^+^CD4^+^ or CD3^+^CD8^+^ cells from uninfected mice were considered as naïve controls. Graphs show the mean ± SD of two independent experiments (n=5). Mann-Whitney U-test, where *p<0.05; **p<0.01. #n/a, not applicable.

To analyze expression differences in chemokines and inhibitory receptors between CXCR5^+^, CXCR5^+^TIM-3^+^ and TIM-3^+^ subsets within both CD4^+^ and CD8^+^ Tex cells during chronic infection, a FACS analysis was performed on freshly isolated dLNs cells after 90 days of infection. With regard to chemokines receptors, both CD4^+^CXCR5^+^ and CD8^+^CXCR5^+^ cells expressed significantly lower levels of lymphoid tissue-homing CXCR5 receptor, compared to their corresponding CXCR5^+^TIM-3^+^ subsets (CD4^+^CXCR5^+^ MFI 1658 vs. CD4^+^CXCR5^+^TIM-3^+^ MFI 2475); (CD8^+^CXCR5^+^ MFI 1214 vs. CD8^+^CXCR5^+^TIM-3^+^ MFI 1510). As expected, TIM-3^+^ cells did not show CXCR5 expression (**Figure 4A**). CD4^+^CXCR5^+^ (MFI 3594) and CD8^+^CXCR5^+^ (MFI 3515) cells showed elevated levels of T-cell zone homing CCR7 receptor, as compared to their corresponding CXCR5^+^TIM-3^+^ (CD4^+^ MFI 3021; CD8^+^ MFI 2677) and TIM-3^+^ (CD4^+^ MFI 2451; CD8^+^ MFI 2623) subsets. No significant differences in CCR7 expression were observed between CXCR5^+^TIM-3^+^ and TIM-3^+^ cells (**Figure 4B**). Significantly decreased levels of peripheral homing CX3CR1 receptor were observed on CD4^+^CXCR5^+^ and CD8^+^CXCR5^+^ cells with respect to their TIM-3^+^ counterparts (CD4^+^CXCR5^+^ MFI 1540 vs. CD4^+^TIM-3^+^ MFI 2074); (CD8^+^CXCR5^+^ MFI 1870 vs. CD8^+^TIM-3^+^ MFI 2204). Intriguingly, the CXCR5^+^TIM-3^+^ subsets within both CD4^+^ and CD8^+^ T-cell compartments displayed the highest expression of CX3CR1 (CD4^+^ MFI 2742; CD8^+^ MFI 3156) (**Figure 4C**). These data collectively indicate that the CXCR5^+^ subsets predominantly express CXCR5 and CCR7 compared to TIM-3 cells, suggesting that the higher expression of these chemokine receptors promotes the homing of CXCR5^+^ cells to the T-cell areas of lymphoid tissues. Additionally, these results also indicate that the CXCR5^+^TIM-3^+^ subsets might preferentially migrate to inflamed tissues as they express the highest levels of CX3CR1.

The analysis of inhibitory receptors revealed that the CD4^+^TIM-3^+^ and CD8^+^TIM-3^+^ subsets exhibited significantly increased levels of the PD-1 exhaustion marker, as compared to their respective CD4^+^CXCR5^+^ (TIM-3^+^ MFI 13938 vs. CXCR5^+^MFI 10956) and CD8^+^CXCR5^+^ (TIM-3^+^ MFI 8776 vs. CXCR5^+^ MFI 1134) counterparts. A sharp rise of PD-1 expression on CXCR5^+^TIM-3^+^ cells within both the CD4^+^ (MFI 41389) and CD8^+^ (MFI 15258) T-cell compartments was observed during the chronic infection. Interestingly, PD-1 expression differences between CXCR5^+^ and TIM-3^+^ subsets were markedly higher in the CD8^+^ T-cell compartment (**Figure 4D**). No significant differences in TIM-3 inhibitory receptor were observed between CD4^+^CXCR5^+^TIM-3^+^ and CD4^+^TIM-3^+^ cells. However, the CD8^+^CXCR5^+^TIM-3^+^ (MFI 4596) subset showed significantly elevated levels of TIM-3 expression compared to the CD8^+^TIM-3^+^ (MFI 3541) subset. As expected, CXCR5^+^ cells did not show TIM-3 expression (**Figure 4E**). CXCR5^+^TIM-3^+^ (CD4^+^ MFI 8951; CD8^+^ MFI 20146) cells exhibited the highest expression of ectonucleotidase CD39, followed by TIM-3^+^ (CD4^+^ MFI 7090 4; CD8^+^ MFI 7747) and CXCR5^+^ (CD4^+^ MFI 3818; CD8^+^ MFI 4846) cells (**Figure 4F**). These data together indicate that CXCR5^+^ cells are phenotypically less exhausted than their CXCR5^+^TIM-3^+^ and TIM-3^+^ counterparts, as they present lower expression of PD-1, TIM-3 and CD39, suggesting that CXCR5^+^ subsets exhibit a less-differentiated state of T-cell exhaustion.

### 3.5 CXCR5^+^ and CXCR5^+^TIM-3^+^ subsets display higher proliferation capacity

**Figure 5:**
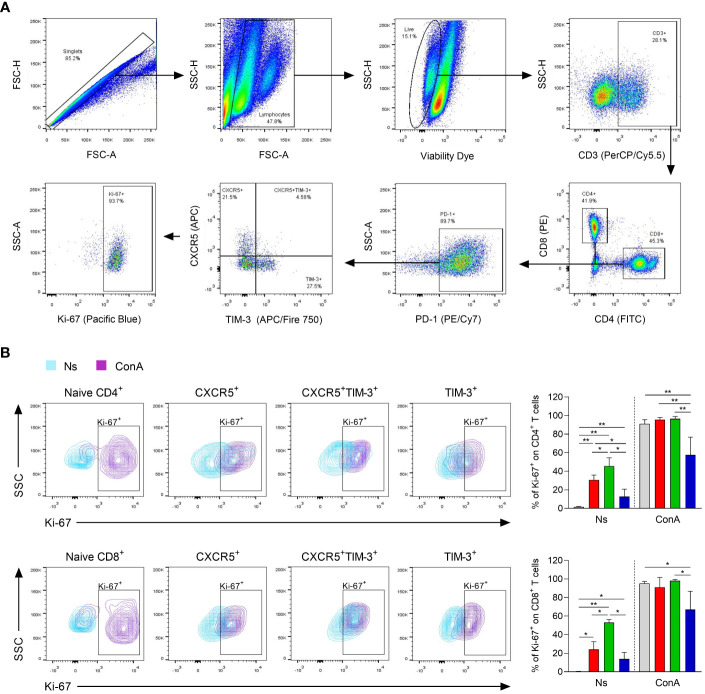
Proliferation capacity of CXCR5^+^, CXCR5^+^TIM-3^+^ and TIM-3^+^ subsets during the chronic phase of *L. mexicana* infection. Popliteal draining lymph node cells derived from chronically infected mice (90 days after infection) were stimulated with ConA (5 μg/mL) for 96 h. **(A)** Gating strategy of Ki-67^+^ cells within Tex subsets of both CD4^+^ and CD8^+^ T cells. **(B)** Representative contour plots and summary data showing Ki-67 frequency (%) of Tex subsets within CD4^+^PD-1^+^ (upper panel) and CD8^+^PD-1^+^ (lower panel) T-cell compartments. CD3^+^CD4^+^ or CD3^+^CD8^+^ cells from uninfected mice were considered as naïve controls. The data represent two independent experiments and are presented as the mean value ± SD (n=5). Mann-Whitney U-test, where *p<0.05; **p<0.01. Ns, non-stimulated; ConA, Concanavalin A.

To determine the ability of the different Tex subsets to proliferate during chronic infection, Ki-67 expression was assessed in response to polyclonal stimulation after 90 days of infection. Ki-67^+^ cells within CXCR5^+^, CXCR5^+^TIM-3^+^ and TIM-3^+^ subsets were gated from the living cells after 96 h of stimulation with or without ConA (**Figure 5A**). After *in vitro* culture without stimulation, the CXCR5^+^ subset within both CD4^+^ and CD8^+^ T-cell compartments exhibited significantly higher frequencies of Ki-67^+^ cells, compared to their respective TIM-3^+^ counterparts. Specifically, the CD4^+^CXCR5^+^ and CD8^+^CXCR5^+^ subsets exhibited approximately 24-30% of Ki-67^+^ cells, whereas the CD4^+^TIM-3^+^ and CD8^+^TIM-3^+^ subsets showed 13-14%. Notably, CXCR5^+^TIM-3^+^ cells within both CD4^+^ and CD8^+^ T cells demonstrated the highest frequencies of Ki-67^+^ cells, reaching values of 45-53% (**Figure 5B**). Furthermore, upon polyclonal stimulation, there was a remarkable increase in the frequency of Ki-67^+^ cells on CXCR5^+^ and CXCR5^+^TIM-3^+^ subsets of both CD4^+^ and CD8^+^ T-cell compartments, with frequencies reaching up to 98%. There was also an increase of 57-67% in the frequencies of Ki-67^+^ cells in the TIM-3^+^ subset of CD4^+^ and CD8^+^ T cells upon ConA stimulation. However, these percentages were not as high as those observed in their respective CXCR5^+^ or CXCR5^+^TIM-3^+^ subgroups (**Figure 5B**). Additionally, the CXCR5^+^ and CXCR5^+^TIM-3^+^ subsets within both CD4^+^ and CD8^+^ T-cell compartments exhibited significantly higher Ki-67 MFI compared to their respective TIM-3^+^ subsets under both unstimulated and ConA-stimulated conditions (**Supplementary Figure 2**). Taken together, these data indicate that a higher percentage of CXCR5^+^ and CXCR5^+^TIM-3^+^ cells display an active cell-cycle progression, suggesting that Tex cells expressing CXCR5 are actively proliferating cells during chronic infection with *L. mexicana*.

### 3.6 CXCR5^+^ subsets exhibit an enhanced capacity to produce IFN-γ, whereas CXCR5^+^TIM-3^+^ and TIM-3^+^ cells show a higher cytolytic potential

**Figure 6:**
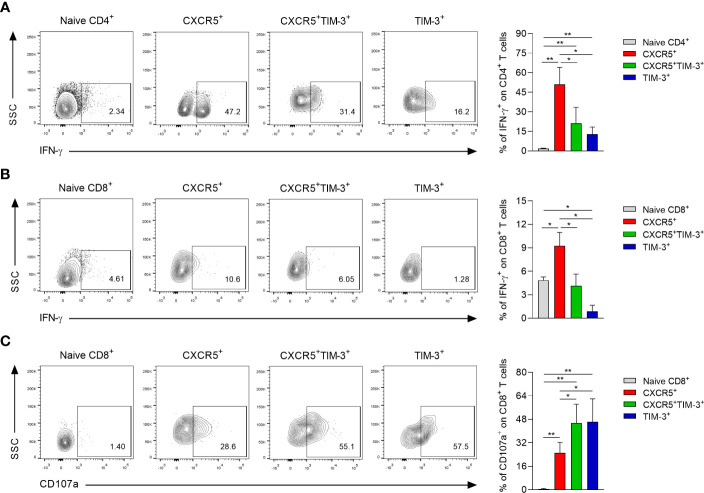
Functional capacity of CXCR5^+^, CXCR5^+^TIM-3^+^ and TIM-3^+^ subsets during the chronic phase of *L. mexicana* infection. Popliteal draining lymph node cells derived from chronically infected mice (90 days after infection) were stimulated with PMA (50 ng/mL) and Ionomycin (1 μg/mL) for 2 h followed by 4 h of incubation with monensin (2 μM). Representative contour plots and summary data showing the IFN-γ **(A, B)** and CD107a frequency **(C)** of Tex subsets within CD4^+^PD-1^+^ and CD8^+^PD-1^+^ T-cell compartments. CD3^+^CD4^+^ or CD3^+^CD8^+^ cells from uninfected mice were considered as naïve controls. Data are representative of two independent experiments and are presented as the mean ± SD (n=5). Mann-Whitney U-test, where *p<0.05; **p<0.01.

To assess the effector function of each Tex subset during chronic infection with *L. mexicana*, IFN-γ and CD107a expression were analyzed by flow cytometry after 90 days of infection. The *in vitro* functional analysis indicated that CXCR5^+^ cells within both CD4^+^ and CD8^+^ Tex compartments exhibited a significantly enhanced capacity to produce IFN-γ upon polyclonal stimulation, whereas the TIM-3^+^ subsets displayed the lowest capacity (**Figures 6A, B**). Notably, CD4^+^CXCR5^+^ Tex cells showed the highest percentage of IFN-γ producing cells (51%), followed by CXCR5^+^TIM-3^+^ (21.1%) and TIM-3^+^ (12.7%) cells, both also within CD4^+^ Tex compartment (**Figure 6A**). In addition, data indicated that the three subsets of exhausted CD8^+^ T cells exhibited a lower capacity to produce IFN-γ in response to PMA/Ionomycin stimulation, as compared to either CD4^+^ Tex cells (**Figure 6B**). Further analysis showed that a greater proportion of CXCR5^+^TIM-3^+^ and TIM-3^+^ cells within the CD8^+^ Tex compartment were positive for the degranulation marker CD107a (45.5% and 46.3%, respectively), whereas the CXCR5^+^ subset displayed the lower percentage of CD107a^+^ cells (25%) (**Figure 6C**). Overall, these results demonstrate distinct functional characteristics between the Tex subsets during chronic infection with *L. mexicana*. Accordingly, CXCR5^+^ cells show an enhance capacity to produce IFN-γ, whereas CXCR5^+^TIM-3^+^ and TIM-3^+^ cells show an increased cytolytic capacity.

### 3.7 Lesion sites exhibit a greater infiltration of TIM-3^+^ cells during the chronic infection

**Figure 7:**
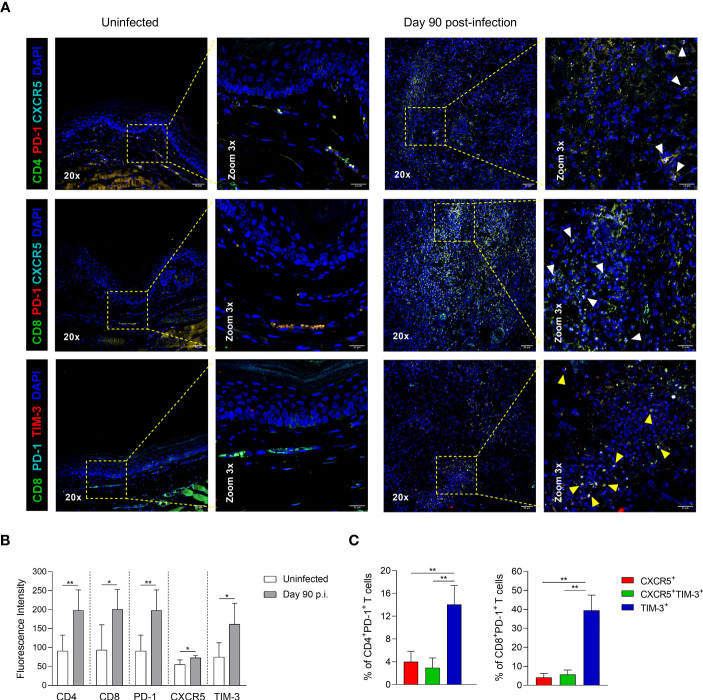
CD4^+^ and CD8^+^ Tex subsets in footpad lesions during chronic *L. mexicana* infection. **(A)** Representative multiplex immunofluorescence images of CXCR5^+^ and TIM-3^+^ cells in footpad tissue sections of uninfected or chronically infected mice at day 90 post-infection. White arrowheads point to PD-1^+^CXCR5^+^ cells, and yellow arrows points to PD-1^+^TIM-3^+^ cells. **(B)** Fluorescence intensity of CD4, CD8, PD-1, CXCR5 and TIM-3 in uninfected or infected paw tissue sections at day 90 of infection. Data represent the mean value ± SD (n=3). **(C)** Frequency of Tex subsets within CD4^+^PD-1^+^ and CD8^+^PD-1^+^ T-cell compartments by flow cytometry at 90 days after infection. Data show the mean value ± SD of two independent experiments (n=5). Mann-Whitney U-test, where *p<0.05; **p<0.01.

To identify the Tex subsets in the infection sites, immunofluorescence preparations of footpad lesions from chronically infected mice (90 days p.i.) were stained to detect the co-expression of PD-1, CXCR5 and TIM-3 in T lymphocytes. Multiplex immunofluorescence revealed that PD-1 and CXCR5 or PD-1 and TIM-3 co-localize with some labeled CD4^+^ or CD8^+^ T cells, indicating the presence of CXCR5^+^ and TIM-3^+^ subsets in the inflammatory infiltrate of chronic lesions (**Figure 7A**). Quantitative fluorescence intensity analysis revealed significantly higher expression levels of CD4, CD8, PD-1, CXCR5, and TIM-3 in the lesion sites at 90 days p.i., compared to uninfected controls. Interestingly, infected tissues showed a 1.3-fold increase in CXCR5 expression and a 2.1-fold increase in TIM-3 expression compared to uninfected controls, suggesting a higher infiltration of TIM-3^+^ cells during chronic infection (**Figure 7B**). Flow cytometry analysis of Tex subpopulations at day 90 of infection further confirmed the predominance of TIM-3^+^ cells within both CD4^+^ and CD8^+^ T-cell compartments in the lesion sites, compared to their respective CXCR5^+^ and CXCR5^+^TIM-3^+^ cells. Notably, the frequency of TIM-3^+^ cells was higher in the CD8^+^ T cell compartment, reaching frequencies around of 40% (**Figure 7C**). These findings collectively demonstrate the enrichment of TIM-3^+^ cells at the infection sites during the chronic phase of the infection.

## 4 Discussion

The term ‘T-cell exhaustion’ was originally used to describe a dysfunctional or non-responsive state resulting from chronic antigen stimulation (13–15). However, emerging evidence has shown that Tex cells can still play an active role in counteracting viral infections and tumor growth, as they comprise a heterogeneous population of progenitor-like and terminally-differentiated cells that maintain some key functional properties (20). Although previous studies have investigated T-cell exhaustion during chronic leishmaniasis, our study is the first to examine the phenotypical and functional heterogeneity of both CD4^+^ and CD8^+^ Tex cells during experimental CL caused by *L. mexicana*.

To investigate T-cell exhaustion in experimental CL, we employed a subcutaneous infection model using *L. major*-resistant C57BL/6 mice infected with an intermediate-dose (10^5^) of *L. mexicana* promastigotes. This particular mouse strain was chosen since they better mimic the natural cutaneous infection and elicit a chronic disease characterized by the persistence of parasites within lesions and impaired immune response, as observed in patients with DCL (30, 31). A follow-up of 90 days showed a progressive development of chronic, non-healing cutaneous lesions with inefficient control of intralesional parasites. These outcomes might be related to the marked reduction of granulocytes and lymphocytes observed in the infection sites and the lack of IFN-γ production in dLNs. Previous studies have shown that susceptibility of C57BL/6 mice to *L. mexicana* infection is linked to a decreased expansion, recruitment and differentiation of T lymphocytes into IFN-γ producing cells (32, 33). Indeed, IFN-γ production is a crucial mechanism for the immunological control of *Leishmania* infection as it leads to the activation of inducible nitric oxide synthase (iNOS) and subsequent NO production within host cells (5). It has been previously demonstrated that IFN-γ and iNOS knockout C57BL/6 mice infected with *L. mexicana* develop exponentially growing lesions with uncontrolled parasite replication. Regarding the latter, several clinical studies have also shown that a predominant Th1 response is associated with the healing of cutaneous lesions (34, 35). Interestingly, Buxbaum et al. (33) found that antigen-specific IFN-γ production in dLNs was remarkably lower in *L. mexicana*-infected C57BL/6 mice compared to mice infected with *L. major*, suggesting an impairment of the T-cell inflammatory response during *L. mexicana* infection. Our current data also showed a significant reduction of IFN-γ production during the chronic phase of the infection. By contrast, during the early infection stage, high levels of this cytokine were observed after antigen restimulation. The reduction on IFN-γ production could be attributed to a decrease in immunodominant antigen-specific T cells and the antagonism between Th1 and Th2 responses. In this regard, it has been previously demonstrated that peripheral deletion of virus-specific CD8^+^ T cells upon prolonged antigen stimulation depends on epitope specificity of T-cell receptors (36).

Susceptibility to leishmaniasis has been extensively associated with anti-inflammatory cytokines, as demonstrated in IL-4, IL-6, IL-10 and IL-13 deficient BALB/c mice (37–40). These cytokines are able to antagonize Th1-type immune responses, attenuate lymphocyte recruitment into inflamed tissues, suppress antigen presentation, inhibit the microbicidal mechanisms of macrophages, and downregulate the synthesis of several pro-inflammatory cytokines (41–43). Here, we now show that dLNs cells of *L. mexicana*-infected mice produce high levels of anti-inflammatory cytokines in response to promastigote antigens. However, the production levels were not as high as expected during the chronic infection, considering lesion sizes and parasite burdens. Surprisingly, we found that IL-4, IL-5, IL-6, IL-10, and IL-13 levels reached peaks after 30 days of infection and then decreased in a time-dependent manner. Our findings are consistent with previous data showing that C57BL/10 mice infected with *L. amazonensis*, a closely related species to *L. mexicana*, express low or lack mRNA levels of IL-4, IL-10, and TGF-β in lymph nodes during the late phase of the infection (44). Similarly, Ji et al. (45) showed that IL-4 production by dLNs cells derived from *L. amazonensis*-infected C57BL/6 mice decreased over the course of the infection. This paradoxically low production of anti-inflammatory cytokines in experimental infections caused by parasites of the *L. mexicana* complex could be explained, at least in part, by the absence of antigen-specific T cells that produce anti-inflammatory cytokines in lymph nodes. Considering that during *Leishmania* infections, activated T cells enter the infection sites regardless of their antigen specificity (46), we speculate that anti-inflammatory T cells migrate from dLNs to footpad lesions as infections caused by species of the *L. mexicana* complex progress to chronic stages, creating an immune privileged site for parasite replication in C57BL/6 mice. This is supported by clinical data showing that non-healing lesions of patients with LCL and DCL are associated with high mRNA *in situ* expression of IL-4, IL-5, IL-10, and TGF-β (34, 47).

The regulation of T-cell responses to chronic or persistent infections involves a dynamic balance between effector functions, required for pathogen elimination, and immunosuppressive mechanisms that limit the unwanted tissue damage (48). *Leishmania* parasites take advantage of these inhibitory mechanism, such as the PD-1/PD-L1 pathway, to successfully persist within vertebrate hosts by dampening antigen-dependent immune activation (49, 50). In the present study, we found that CD4^+^PD-1^+^ and CD8^+^PD-1^+^ T cells continuously increase in dLNs over the time course of the *L. mexicana* infection. Furthermore, we also found increasing levels of PD-1 expression on both CD4^+^ and CD8^+^ T cells, indicating that chronic non-healing lesions are related to T-cell exhaustion. It has been proposed that the degree of exhaustion is associated with disease severity (51). In leishmaniasis, this is supported by a previous study showing that CD8^+^ T cells from patients with DCL exhibit functional exhaustion, as demonstrated by defective proliferation and impaired functional activity (7). Our results now clearly show that Tex cells in dLNs increase proportionally to lesion size, suggesting that PD-1 expression might be related to parasite load. In support, higher frequencies of PD-1^+^ cells within different CD4^+^ T cell subsets have been reported in patients with active cutaneous lesions as compared to post-treatment patients (52). It is well known that persistent T-cell receptor stimulation by high antigen concentrations induces fixed epigenetic modifications in the *Pdcd1* locus, resulting in the maintenance of high levels of PD-1 expression on antigen-specific T cells (53). In this regard, Martinez Salazar et al. (54) indicated that PD-1 up-regulation on CD8^+^ T cells is related to parasite load, as shown in BALB/c mice infected with two different doses of *L. mexicana* promastigotes. It is noteworthy that our results showed elevated numbers of PD-1^+^ cells, as well as enhanced PD-1 expression, in the CD4^+^ T-cell compartment of C57BL/6 mice. Therefore, it is tempting to speculate that susceptibility to *L. mexicana* infection in this mouse strain might be mainly related to CD4^+^ T-cell exhaustion. Regarding the latter, previous studies have shown higher expression levels of PD-1 on CD8^+^ T cells of BALB/c mice chronically infected with species of the *L. mexicana* complex (54–56). Additionally, it has recently been demonstrated that the treatment of *L. amazonensis*-infected BALB/c mice with either anti-PD-1 or anti-PD-L1 mAbs results in a high reinvigoration of CD8^+^ T-cell activity, leading to the control of parasite load (56).

A significant advancement in the understanding of T-cell exhaustion was the discovery that Tex cells represent a heterogeneous group, involving cells with different phenotypes with varying transcriptional as well as functional characteristics (20). We identified, for the first time to the best of our knowledge, three subpopulations of Tex cells within both CD4^+^PD-1^+^ and CD8^+^PD-1^+^ compartments during experimental cutaneous infection by *L. mexicana*, including CXCR5^+^, CXCR5^+^TIM-3^+^ and TIM-3^+^ subsets. Similar subpopulations have been reported in chronic viral infections and in several types of cancers in both human and animal models. For instance, at least two major PD-1-expressing subsets have been identified as TCF1^+^ progenitor-like cells with memory/stem like properties, and terminally-differentiated cells that represent the so-called exhausted cells, characterized by limited proliferation and diminished effector functions (21–29). According to our phenotypical and functional analysis, we speculate that the CXCR5^+^ subset might resemble TCF1^+^ progenitor-like cells, which is in accordance with the report that the TCF1/Bcl6 axis positively regulates the CXCR5 expression on CD8^+^ T cells during chronic infections (23). Conversely, CXCR5^+^TIM-3^+^ and TIM-3^+^ subsets might be more similar to terminally-differentiated cells due to their higher expression of inhibitory receptors (21–23). Taking this into account, we hypothesize that a similar differentiation program of Tex subsets occurs after sustained antigen stimulation, independent of the disease-specific milieu. In this regard, Miller et al. (28) showed that chronic lymphocytic choriomeningitis virus infection (LCMV-Cl13) and B16.F10 melanoma tumors elicit analogous subsets of CD8^+^ Tex cells, which share transcriptional, phenotypic, functional, and epigenetic properties. Further research is needed to determine if there is a similar pattern between the Tex subsets identified in our study and the subsets previously reported in other models of chronic infections or malignances.

Our longitudinal analysis of lymph nodes revealed that the CXCR5^+^ subpopulation augmented as infection progressed, whereas both CXCR5^+^TIM-3^+^ and TIM-3^+^ subsets increased progressively during the first 60 days, and thereafter decreased during the remaining 90 days post-infection. In contrast to lymph nodes, we observed a high infiltration of TIM-3^+^ cells within the footpad lesions at day 90 of infection. Collectively, these observations suggest that lymph nodes are the primary location of CXCR5^+^ cells throughout the course of the infection, whereas CXCR5^+^TIM-3^+^ and TIM-3^+^ cells migrate to peripheral tissue during the chronic phase. In support, studies have shown that CXCR5-expressing CD8^+^ T cells are found predominantly in secondary lymphoid organs, while terminally-differentiated cells are localized in major infection sites, including lymphoid and non-lymphoid tissues (21, 22). It is noteworthy that CXCR5^+^ cells continued to progressively increase in the lymph nodes draining the infection site as the infection time progressed. This observation is quite different from early reports of viral infections, showing that the frequency of CXCR5^+^TIM-3^-^ CD8^+^ T cells rapidly increases during the first days of chronic infection, maintaining high and constant levels through viremia (21). It has been documented that CXCR5^+^TIM-3^-^ CD8^+^ T cells exhibit greater therapeutic potential against chronic viral infections, as shown by adoptive transfer experiments (22).

Based on our current evidence, we speculate that the increasing number of intralesional parasites is associated with the continuous increase of CXCR5^+^ cells in dLNs, prompting the idea that these cells play a role in experimental CL. Although our current results do not clarify this underlying role of CD4^+^CXCR5^+^ and CD8^+^CXCR5^+^ cells during *L. mexicana* infection, we found that dLNs of chronically infected mice with a low-pathogenic strain of *L. mexicana*, isolated from a patient with LCL, had a higher frequency of CXCR5^+^ subsets compared to mice infected with a high-pathogenic strain or strain isolated from a DCL patient (**Supplementary Figure 3**). These results provide evidence that CXCR5^+^ cells might be associated with the development of smaller cutaneous lesions.

The current knowledge of exhausted subsets indicates that TCF1^+^ progenitor-like CD8^+^ T cells reside in a specialized niche within lymphoid tissues or tertiary lymphoid structures (TLEs) to retain their memory/stem cell-like characteristics under conditions of long-term chronic infections or tumorigenesis (21, 57, 58). This notion is consistent with our data showing that CXCR5^+^ cells express higher levels of lymphoid tissue-homing receptors compared to their TIM-3^+^ counterparts during chronic *L. mexicana* infection. It is widely recognized that immune effector cells expressing PD-1 migrate from the blood to the sites of immune priming in secondary lymphoid organs or tumor microenvironment via CXCR5/CXCL13, in which they become resident cells (58). Regarding the latter, Im et al. (57) demonstrated that TCF1^+^CXCR5^+^ CD8^+^ T cells are detectable in blood for at least 15 days of LCMV-Cl13 infection, and thereafter they are found exclusively in the spleen of infected mice. On the other hand, CCL19/CCL21-CCR7 axis plays an important role in positioning lymphocytes within the T-cell zones of lymphatic organs, where they interact with dendritic cells for activation and differentiation (59). Given that we found enhanced levels of CCR7 in the CXCR5^+^ subset within both CD4^+^ and CD8^+^ T-cell compartments compared to CXCR5^+^TIM-3^+^ and TIM-3^+^ cells, we speculate that these cells are mostly localized in T-cell zones despite the high expression of CXCR5. In support, previous data have shown that CXCR5-expressing CD8^+^ T cells are predominantly situated in T-cell zones rather than B-cell areas and are able to migrate in response to CCL19/21 (21).

Our phenotypical characterization also revealed that CXCR5^+^TIM-3^+^ cells express the highest levels of CX3CR1, suggesting that this subset mainly migrates to footpad lesions. In this regard, it has been shown that a fraction of newly generated CXCR5^+^TIM3^-^ cells expressing CX3CR1 egress from lymphoid organs, enter to circulation and migrate to inflamed tissues, where they eventually differentiate into terminally exhausted cells due to excessive antigen stimulation (26, 27). This idea aligns with our findings showing that non-healing footpad lesions exhibit a greater infiltration of TIM-3^+^ cells during the chronic phase of the infection. Further studies are needed to better understand the chemotactic migration patterns and anatomical location of Tex subsets during CL. Moreover, the use of alternative markers to identify progenitor-like cells in the footpad lesions of infected mice should be considered, since a previous study has reported that TCF1-expressing cells located within tumor tissue lack the expression of CXCR5 (28).

Interestingly, our data not only showed a pattern of lymphoid tissue-homing receptors in the CXCR5^+^ subset during chronic infection that supported their localization in lymph nodes, but also demonstrated that this subpopulation displays a less exhausted phenotype compared to their TIM-3^+^ counterpart. In line with this, we found that CXCR5^+^ cells within both CD4^+^ and CD8^+^ T-cell compartments exhibited lower or no expression of PD-1, TIM-3, and CD39. This observation is consistent with previous reports showing that CXCR5-expressing CD8^+^ T cells exhibit lower levels of several exhaustion-associated markers, including PD-1, TIM-3, LAG-3, 2B4, CD160, and CD39 (21–23). It has been demonstrated that the functional impairment of Tex cells correlates with the number and intensity expression of inhibitory receptors, which act synergistically via non-redundant signaling pathways to establish T-cell exhaustion (60). This notion is consistent with our functional assays, which showed that the frequency of Ki-67 and IFN-γ expressing cells was strikingly higher in the CXCR5^+^ subset of both CD4^+^ and CD8^+^ T-cell compartments, compared to their respective TIM-3^+^ subsets. Additionally, we observed higher expression levels of CD107a in CD8^+^TIM-3^+^ cells, suggesting a predominant cytotoxic phenotype in these cells. It has been shown that TCF1 expression preserves the proliferative potential of T cells, whereas B lymphocyte-induced maturation protein 1 (Blimp1), previously reported in terminally-differentiated cells, upregulates cytotoxic molecules expression and downregulates genes associated with T-cell memory, including *Il7ra*, *Ccr7*, *Cxcr5*, and *Sell* (25).

Our findings raise the possibility that CXCR5^+^ cells could exert some control over leishmaniasis, as we observed that these cells retain their ability to proliferate and produce IFN-γ in response to polyclonal stimulation. It has been demonstrated that anti-viral and anti-tumor immunity after PD-1/PD-L1 checkpoint blockade is mediated by the proliferative burst of TCFI^+^CXCR5^+^ cells (21, 22, 27–29). Although our results do not elucidate the lineage relationship between the Tex subsets, evidence indicates that progenitor-like CD8^+^ T cells differentiate into highly proliferative and cytotoxic transitory states that directly eliminate virus-infected or tumor cells (26, 27). In line with this principle, we observed that both CD4^+^CXCR5^+^TIM-3^+^ and CD8^+^CXCR5^+^TIM-3^+^ cells displayed the highest frequencies of Ki-67^+^ cells. Furthermore, CD8^+^CXCR5^+^TIM-3^+^ showed an elevated expression of CD107a. These results imply that CXCR5^+^TIM-3^+^ cells could also contribute, directly or indirectly, to intralesional parasite elimination before differentiating into TIM-3^+^ cells, possibly reaching the non-lymphoid infected tissues through the CX3CL1/CX3CR1 axis. The proliferation and effector functions of CXCR5^+^ and CXCR5^+^TIM-3^+^ subsets in response to *Leishmania* antigens should be evaluated to determine the presence of antigen-specific cells.

Unexpectedly, we found comparable patterns of tissue distribution, surface expression and functional activity between the analogous exhausted subsets of CD4^+^ and CD8^+^ T cells during the infection, suggesting that CD4^+^ Tex cells follow a similar differentiation program as CD8^+^ Tex cells in our chronic infection model by *L. mexicana*. To date, only a few studies have investigated the distinct differentiation states on CD4^+^ Tex cells during chronic infections or tumorigenesis. Balanca et al. (61) showed that head and neck tumor-infiltrating CD4^+^ T lymphocytes (TILs) comprise two exhausted subsets based on ENTPDI expression, which encodes to CD39. Consistent with our results, they found that PD-1^hi^CD39^-^ CD4^+^ T cells with a less exhausted phenotype had a higher cytokine response. On the other hand, Xia et al. (62) identified analogous populations of PD-1^+^TCF1^+^BCL6^lo/-^ progenitor CD4^+^ T cells distinct from effector and follicular helper T cells in chronic LCMV-Cl13 infection and 1956-mOVA sarcoma tumors, which exhibited similarities with progenitor-like CD8^+^ T cells. In addition, Fu et al. (63) reported that treatment with anti-PD-L1 mAb increased TCF-1 and decreased TIM-3 and LAG-3 expression in tumor-specific CD4^+^ cells isolated from the spleen of B16.F10-tumor bearing mice, suggesting that PD-1/PD-L1 immunotherapy also increases TCF1^+^ CD4^+^ Tex cells. Although the vast majority of studies have focused on TCF1^+^ progenitor-like CD8^+^ T cells, these findings highlight the role of TCF1^+^ CD4^+^ Tex cells in response to chronic diseases. Therefore, the CD4^+^CXCR5^+^ subset identified in our study could also have a significant impact on long-term protection against CL due to their enhanced capacity to proliferate and produced IFN-γ. In fact, a previous study demonstrated that the accumulation of CD4^+^CXCR5^+^ T cells capable of producing pro-inflammatory cytokines within TLEs of lungs was associated with immune control of experimental *Mycobacterium tuberculosis* infection (64). Further research is required to determine more specific criteria that delineate the TCF1^+^ progenitor-like CD4^+^ T-cell subset in our infection model, as other populations of CD4^+^ T cells express both TCF1 and CXCR5.

In summary, we have demonstrated that PD-1^+^ Tex cells comprise three major subpopulations based on CXCR5 and TIM-3 expressions in *L. mexicana* infection. Further, we have shown that each subset differs in its phenotype, functional features, and tissue distribution during the chronic phase. Importantly, our data suggest that the less-differentiated CXCR5^+^ Tex subsets might contribute to the control of cutaneous infection, as they retain proliferation capacity and IFN-γ production. Further studies will be necessary to determine if any of the Tex subsets identified in this work play a determinant role in maintaining the immune response during CL, yet the study outlined here provides a foundation to explore the potential therapeutic use of CXCR5^+^ cells against the chronic forms of leishmaniasis.

## Data availability statement

The original contributions presented in the study are included in the article/**Supplementary Material**. Further inquiries can be directed to the corresponding author.

## Ethics statement

The animal study was approved by Comité Interno para el Cuidado y Uso de Animales de Laboratorio, UNAM (Approval No. 123-2019/038-CIC-2019 and 063-2020/022-CIC-2020). The study was conducted in accordance with the local legislation and institutional requirements.

## Author contributions

Conceptualization: MD, LB and IB. Design experiments: MD, JZ-C and IB. Methodology and investigation: MD. Flow cytometry acquisition data: JZ-C. Immunofluorescences: JG. Analyze and data interpretation: MD, JG and JZ-C. Supervision: LB and IB. Funding acquisition: IB. Writing, original draft: MD. Review and editing manuscript: JZ-C and IB. All authors contributed to the article and approved the submitted version.
