# Supplementary material for: CXCR5 and TIM-3 expressions define distinct exhausted T cell subsets in experimental cutaneous infection with Leishmania mexicana
Source: Front Immunol. 2023 Aug 25;14:1231836. doi: 10.3389/fimmu.2023.1231836 (PMC10485697; doi:10.3389/fimmu.2023.1231836)
Supplement: Supplementary file 1 [file DataSheet_1.docx]

Supplementary Figures


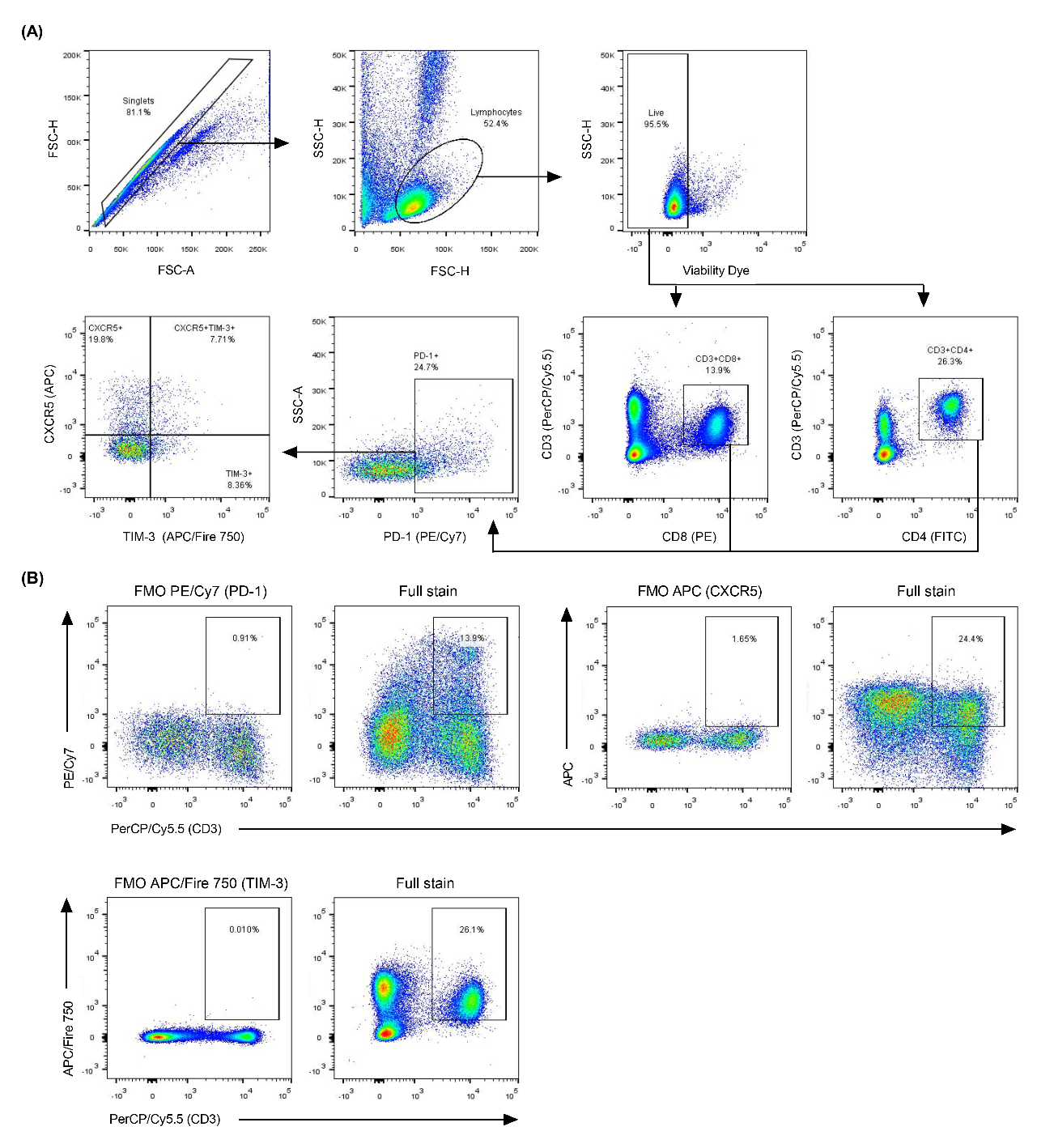


**Supplementary Figure 1.** Gating strategy for Tex subsets identification during *L. mexicana* infection. (A) Doublets were excluded based on the forward scatter-height (FSC-H) vs. forward scatter-area (FSC-A) and total lymphocytes were subsequently gated with side scatter-height (SSC-H) vs. FSC-H. Thereafter, dead cells were excluded by gaiting the viability dye-negative events (live) and then separated into CD3^+^CD4^+^ and CD3^+^CD8^+^. Both T-cell compartments were gated further into PD-1^+^ events and subsequently fractionated into CXCR5^+^, CXCR5^+^TIM-3^+^ and TIM-3^+^ subsets. (B) Fluorescence-minus-one (FMO) controls for the fluorochromes conjugated with PD-1, CXCR5 and TIM-3. For each, the graphs are presented in pairs, with the FMO control displayed on the left and full stain on the right. Tex subsets from both CD4^+^ and CD8^+^ T cells were identified by the same gates.


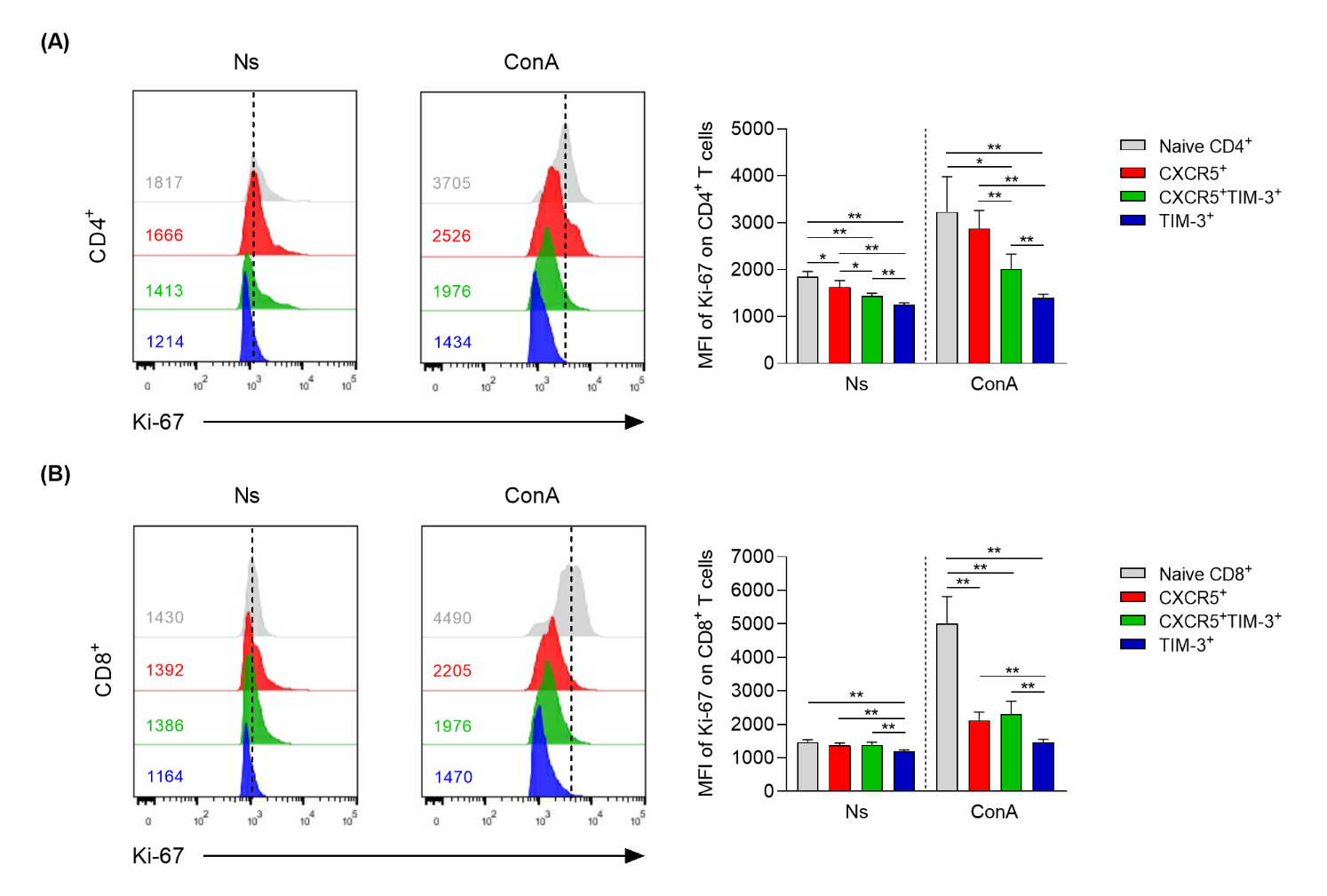


**Supplementary Figure 2.** Ki-67 expression on CXCR5^+^, CXCR5^+^TIM-3^+^ and TIM-3^+^ subsets during the chronic phase of *L. mexicana* infection. Popliteal draining lymph node cells derived from chronically infected mice (90 days after infection) were stimulated with ConA (5 μg/mL) for 96 h. Representative histograms and summary data showing the median fluorescence intensity (MFI) of Ki-67 on Tex subsets within CD4^+^PD-1^+^ (A) and CD8^+^PD-1^+^ (B) T-cell compartments. CD3^+^CD4^+^ or CD3^+^CD8^+^ cells from uninfected mice were considered as naïve controls. Graphs show the mean ± SD of two independent experiments (n=5). Mann-Whitney U-test, where *p<0.05; **p<0.01.


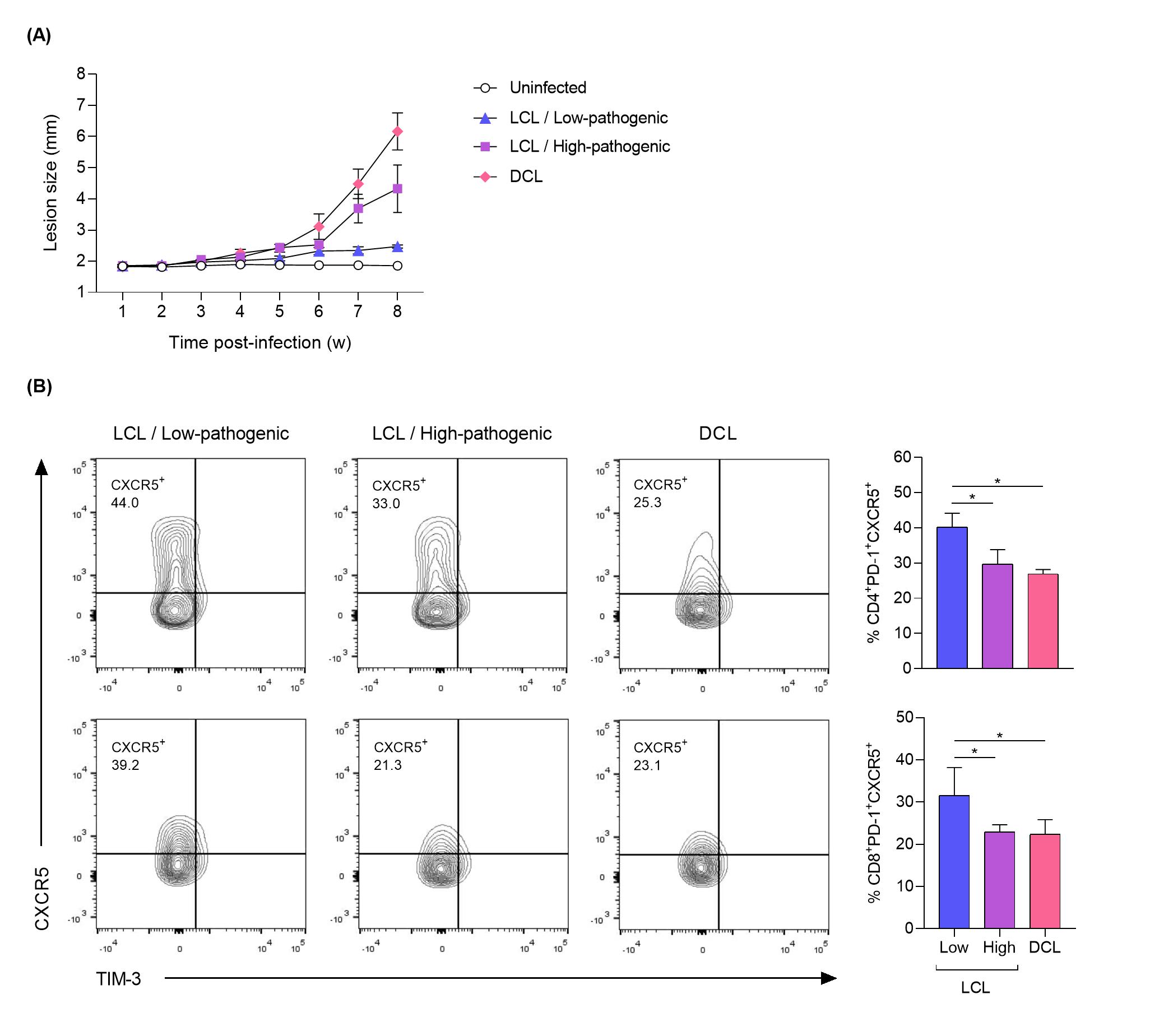


**Supplementary Figure 3.** CXCR5^+^ subset in C57BL/6 mice infected with different *L. mexicana* strains isolated from patients diagnosed with LCL or DCL. (A) Cutaneous lesion development of female C57BL/6 mice subcutaneously infected in the hind footpad with 2x10^6^ stationary-phase promastigotes strains isolated from LCL or DCL patients. Data represent the mean value ± SD (n=3). (B) Representative contour plots and summary data of CXCR5^+^ frequency within both CD4^+^PD-1^+^ and CD8^+^PD-1^+^ T-cell compartments derived from draining lymph nodes of infected mice. Graphs shows the mean ± SD (n=3). Mann-Whitney U-test, where *p<0.05. LCL, localize cutaneous leishmaniasis; DCL, diffuse cutaneous leishmaniasis.
